# Predicting the Degree of Fresh Tea Leaves Withering Using Image Classification Confidence

**DOI:** 10.3390/foods14071125

**Published:** 2025-03-25

**Authors:** Mengjie Wang, Yali Shi, Yaping Li, Hewei Meng, Zezhong Ding, Zhengrui Tian, Chunwang Dong, Zhiwei Chen

**Affiliations:** 1 Tea Research Institute of Shandong Academy of Agricultural Sciences, Jinan 250100, China; chuanyi2023@163.com (M.W.);; 2 College of Mechanical and Electrical Engineering, Shihezi University, Shihezi 832003, China

**Keywords:** fresh tea leaves, moisture content, confidence weighting, degree of withering, YOLOv8

## Abstract

Rapid and non-destructive detection methods for the withering degree of fresh tea leaves are crucial for ensuring high-quality tea production. Therefore, this study proposes a fresh tea withering degree detection model based on image classification confidence. The moisture percentage of fresh tea leaves is calculated by developing a weighted method that combines confidence levels and moisture labels, and the degree of withering is ultimately determined by incorporating the standard for wilted moisture content. To enhance the feature extraction ability and classification accuracy of the model, we introduce the Receptive-Field Attention Convolution (RFAConv) and Cross-Stage Feature Fusion Coordinate Attention (C2f_CA) modules. The experimental results demonstrate that the proposed model achieves a classification accuracy of 92.7%. Compared with the initial model, the detection accuracy was improved by 0.156. In evaluating the predictive performance of the model for moisture content, the correlation coefficients (Rp), root mean square error (RMSEP), and relative standard deviation (RPD) of category 1 in the test set were 0.9983, 0.006278, and 39.2513, respectively, and all performance were significantly better than PLS and CNN methods. This method enables accurate and rapid detection of tea leaf withering, providing crucial technical support for online determination during processing.

## 1. Introduction

In the process of black tea production, withering is the first vital step and the key to making good quality black tea [1,2]. Wilting refers to a series of characteristic changes in the physical parameters of tea leaves, such as moisture content and color, under specific temperature and humidity conditions [3]. As withering progresses, the water content of fresh tea leaves gradually decreases, the leaves become soft, they shrink, their color darkens, and the grassy flavor gradually disappears, resulting in a distinctive fragrance [4,5,6]. Moisture content is an important indicator of the degree of withering of fresh tea leaves. The industry standard for wilting moisture content in fresh tea leaves is 58–66%. In actual production engineering, the degree of withering mainly relies on a combination of manual judgment and leaf moisture content detection. However, manual judgment is easily influenced by factors such as light, personal experience, and subjective judgment, hence it cannot accurately and objectively determine the degree of withering of fresh tea leaves. In addition, such judgments about color and texture are qualitative rather than standardized [7], making it difficult to distinguish subtle differences between colors and textures, which can easily lead to significant errors and inaccurate judgments. As such, traditional detection methods have the weaknesses of low accuracy, lack of objectivity, and slow speed [8].

In recent years, machine vision [9] combined with machine learning has been used effectively to extract image colors and textures for tasks such as image classification, detection and segmentation, achieving efficient image processing and analysis in various fields of agriculture. Wijaya et al. (2023) [10] used electronic noses and optimized machine learning algorithms to effectively evaluate and predict the quality of non-infused tea aroma, achieving the identification of the quality of non-infused Gambung Green Tea. Li et al. (2023) [11] used the least squares support vector machine qualitative model analysis to quickly detect the fermentation degree of Pu’er tea online, achieving a prediction accuracy of 99.3% on the fermentation prediction set. However, in terms of moisture content detection, Zhang et al. (2024) [12] collected near-infrared spectral data in seven stages of green tea processing and established a water content prediction model for green tea using partial least squares regression and backpropagation neural network, achieving high detection performance. Wang et al. (2023) [13] extracted nine color and five texture features from tea images and constructed two preprocessing methods and two optimization algorithms to predict the moisture content of fresh tea leaves through cross combination. However, machine learning detection methods can only input fixed fresh tea features (such as color and texture), which limits the model’s ability to learn features.

Deep learning [14], as a branch of machine learning, is based on the core idea of extracting advanced features of images through multi-level neural networks, thereby achieving tasks such as target classification, recognition, and segmentation. In recent years, deep learning has developed rapidly and applied in various aspects of the tea fields. Chen et al. (2024) [15] proposed an improved YOLOV7 tea bud detection network with multiple sizes and targets in the field of view. By introducing attention mechanism and multi-feature fusion module, an average accuracy of 94.43% was achieved. Based on the regional convolutional neural network Mask-RCNN, Wang et al. (2023) [16] proposed a harvesting point localization method to classify, regress, and segment buds by training regression classification and preselected frames and eliminating the quantization error using regional feature aggregation. An et al. (2020) [17] proposed a withered water detection method based on convolutional neural network confidence to achieve effective detection of moisture content in tea leaves.

The YOLOv8 image classification model, known for its balanced trade-off between speed and accuracy, is utilized as the core algorithm in this study to predict the moisture content of withered fresh tea leaves. A novel weighted method is developed, which combines image classification confidence scores with moisture labels to calculate the percentage of moisture in the tea leaves. This is then correlated with the established standard for wilting moisture content in tea leaves, enabling the determination of the degree of wilting. This approach facilitates rapid, non-destructive detection of the withering level of fresh tea leaves. Furthermore, to enhance both the efficiency and accuracy of the detection process, the model was optimized by sacrificing a small portion of inference speed, significantly improving detection precision. This advancement provides a reliable tool for assessing the quality of tea in subsequent stages of processing.

## 2. Materials and Methods

### 2.1. Image Acquisition and Preprocessing

**Figure 1:**
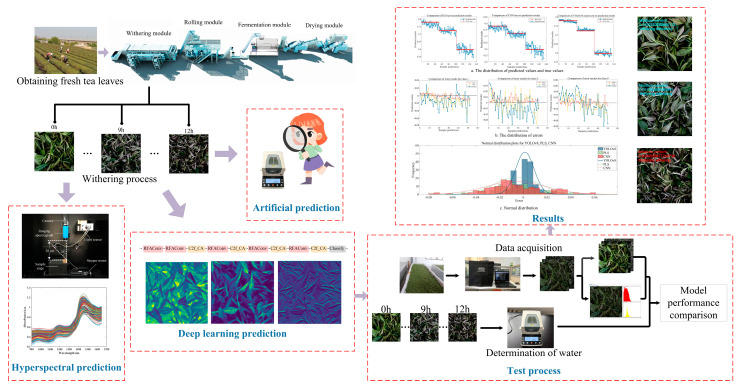
Data collection and algorithm process.

The withered fresh tea leaf images used in this study come from the Tea Research Institute of the Chinese Academy of Agricultural Sciences. The tea variety is Jin Guan Yin, collected in March 2024. In this study, we developed a data collection methodology that involved the synchronized acquisition of both high-resolution images and corresponding moisture measurements across 13 consecutive temporal intervals. Continuous time interval sample data can reflect the impact of changes in moisture content on image color, texture, and other features, helping the model better learn subtle changes in tea phenotype. The data collection and experimental process is shown in Figure 1.

During image acquisition, the acquisition system consists of an artificial climate chamber, an industrial camera (Intel RealSense D405, Intel Corporation, Santa Clara, CA, USA), a uniform light source, and a sample container. During the collection process, the camera is placed 28 cm away from the sample. Samples are collected once every hour, and images of the samples are taken at 13 consecutive time points. A moisture analyzer (XY-110MW) measures the moisture value at each collection time point. Three measurements are recorded at each time point, and the average value is taken as the withering moisture label for that time point. This study obtained a total of 709 wilted fresh tea images and 13 moisture category labels. The data with category labels 1, 5, and 9 were used as an external test set (a total of 153 images). The remaining 10 categories (a total of 556 images) were divided into training sets (a total of 501 images) and validation sets (a total of 55 images) in a ratio of 9:1 to ensure the generalization effect of the model. To prevent the model from overfitting during the training process, a total of 2255 image samples were obtained in this study by cropping, rotating, flipping, and changing the brightness as data enhancement methods of images in the training set.

The experimental data of PLS [18] and CNN [19] were extracted from 709 images using a tea visual image acquisition and analysis software (Software Copyright No. 2014 SR 149549) developed based on Matlab GUI module extracted nine color metrics and six texture features. The nine color metrics include average red component, average green component, average blue component, color angle, average saturation, average luminance component, super-green transformation, ratio of the average red component to average green component, and average hue. The six texture features are average grayscale value, standard deviation, grayscale histogram smoothness, third-order moment bias, grayscale histogram consistency, and grayscale histogram entropy.

**Figure 2:**
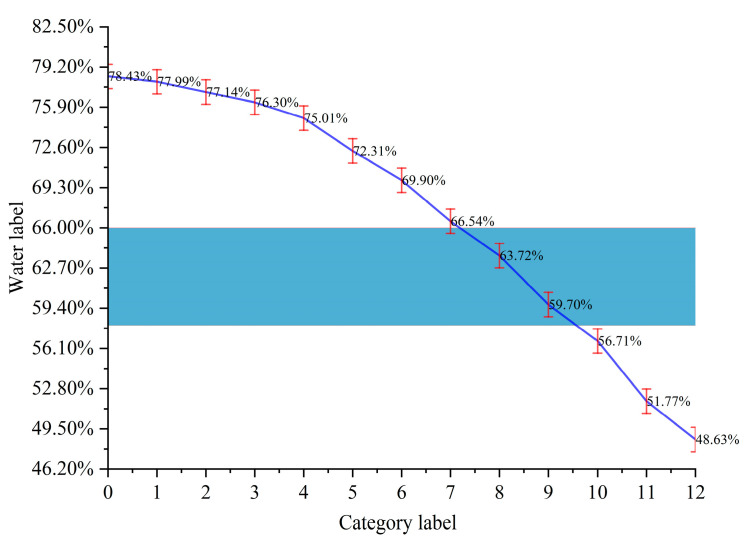
Moisture values of fresh tea leaves under continuous time and the standard for determining the degree of withering.

Figure 2 shows the moisture percentage and the criteria for determining the degree of withering under continuous time. In Figure 2, the blue shadow indicates moderate withering (moisture content between 58% and 66%), the area above the blue shadow indicates insufficient withering (moisture content greater than 66%), and the area below the blue shadow indicates excessive withering (moisture content less than 58%). The coordinates of the intersection point between the fold line and the blue shadow indicate that, at time t, the fresh tea leaves have reached a moderate withering value.

### 2.2. Model Selection and Optimization for Judging the Degree of Withering

#### 2.2.1. Model Selection

YOLOv8 is a major update of YOLOv5 in 2023, which has greatly improved the detection performance of the model [20]. The core of YOLOv8 image classification is to extract surface features from different feature layers of the input image through convolutional layers, then gradually extract abstract features of the image through multiple convolutions, achieving accurate image classification. However, selecting different variants in different application scenarios is the key to maximizing the advantages of YOLOv8. In image classification models, based on increasing network depth and width, the model is divided into five versions: YOLOv8n, YOLOv8s, YOLOv8m, YOLOv8l, and YOLOv8x. The greater the depth, the higher the accuracy and the lower the timeliness. In this study, we selected YOLOv8s as the prediction model for moisture content and improved its basic structure to enhance the prediction accuracy. The results of the models trained with different versions are presented in Section 3.1.

The structure of YOLOv8s is different from that of mainstream image classification models, as it consists of a backbone and head module. The model structure is shown on the left side of Table 1. To enhance the model’s prediction accuracy for the withering degree of tea leaves, this study replaces the standard convolution with the Receptive-Field Attention Convolution (RFAConv) to address the issue of parameter sharing within the model. Additionally, the CA mechanism is incorporated into the C2f module to mitigate the problems of information loss and inaccurate feature fusion during the model’s feature integration process. The improved model results are shown on the right side of Table 1.

##### Perceived Field Attention Convolutional RFAConv

RFAConv is a combination of the RFA attention mechanism and convolution, designed to address the issue of parameter sharing in convolutional kernels [21]. Unlike traditional convolutions, RFAConvs dynamically adjust the receptive field for each input image, enabling the model to more accurately learn local features. The RFA mechanism uses average pooling to capture global information within each receptive field. It then applies a 1 × 1 convolution to facilitate feature interaction. Finally, it uses the Softmax function to weigh the importance of each receptive field. This approach effectively captures spatial feature differences and overcomes the limitations of parameter sharing in convolutional neural networks. The calculation method is detailed in Equation (1).

(1) $$F=Softmax\left(g^{1\times 1}\left(AvgPool\left(X\right)\right)\right)\times ReLU\left(Norm\left(g^{k\times k}\left(X\right)\right)\right)=A_{rf}\times F_{rf}$$

Here, *g*^1×1^ is 1 × 1 grouped convolution, *X* is the input feature map, *Norm* is normalization, *K* is the size of the convolution kernel, *F* represents the product of the attention feature map *A_rf_* and the receptive field spatial feature *F_rf_*.

**Figure 3:**
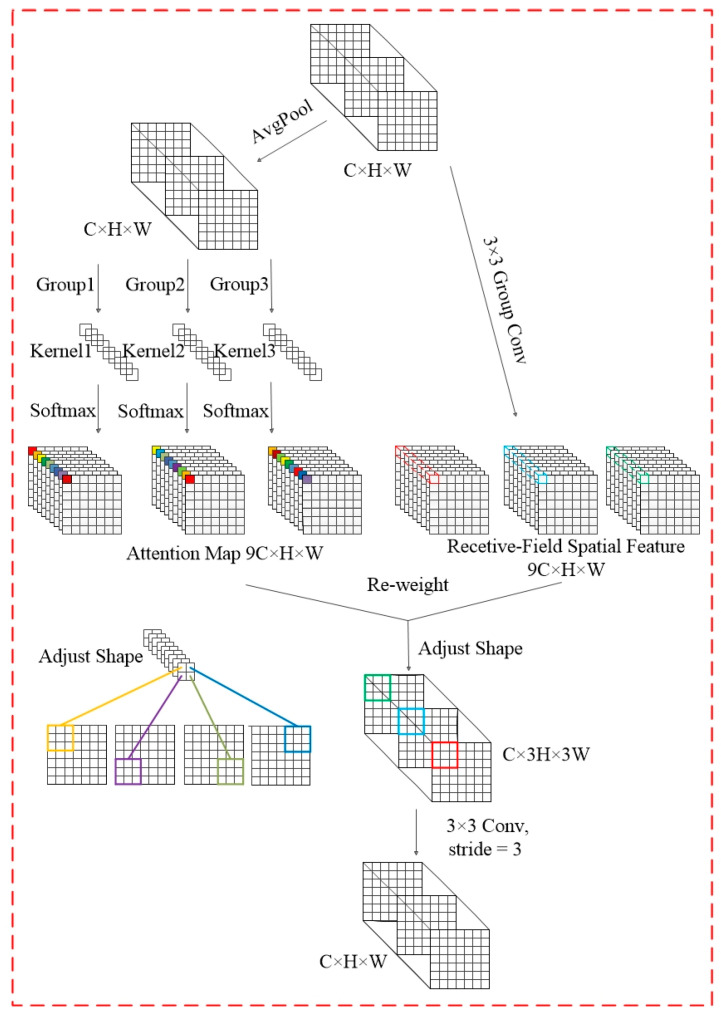
Structure diagram of RFAConv.

In RFAConv implementation, the RFA-generated feature maps are reshaped to eliminate spatial overlap, aggregating all receptive field block features. This allows the model to assign specific convolutional kernels to each receptive field, enhancing its ability to adapt to varying input complexities. The method’s structure is detailed in Figure 3.

##### Cross-Stage Feature Fusion Coordinate Attention

**Figure 4:**
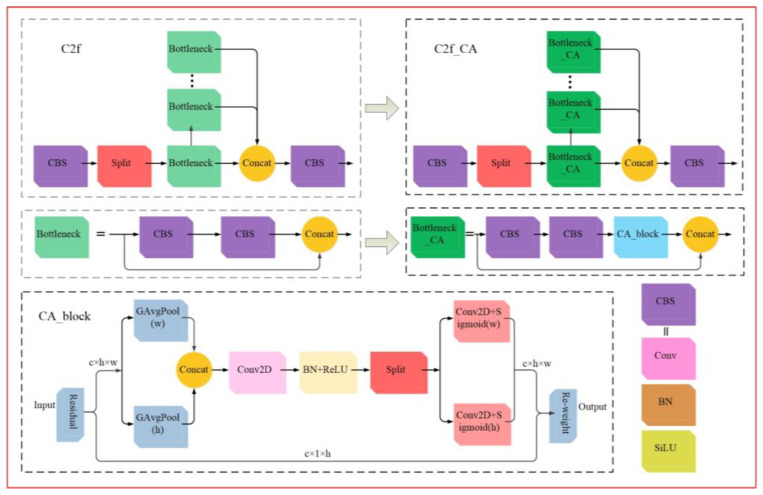
Structural diagram of C2f before and after optimization.

The feature fusion process in the C2f module of the YOLOv8s classification model suffers from minor information loss and inaccuracies. To address this issue, the Coordinate Attention (CA) [22] is introduced into the C2f module, resulting in the development of the Cross-Stage Feature Fusion Coordinate Attention (C2f_CA) module. By utilizing CA-block’s attention to width and height dimensions, the model’s sensitivity to subtle variations in the image is enhanced, leading to more accurate recognition and classification. The C2f_CA module enables the model to extract higher-level information from the feature map, retain more useful feature details, and reduce information loss during the feature transmission process, thereby improving the model’s detection accuracy. The structural diagrams of the module before and after optimization are shown in Figure 4.

### 2.3. Quantitative Prediction of Moisture Content and Determination of Wilting Degree Based on Classification Confidence

In this study, a model is developed by using tea leaf images from the training set as input and moisture content and withering degree as output, as shown in Equation (2). The model outputs predictions for the moisture content and withering degree of tea leaves by performing weighted calculations of image classification confidence and moisture content labels, in conjunction with the standard for tea leaf withering moisture content. The confidence is calculated by measuring the similarity between the input image features and the predicted values. The higher the confidence, the more reliable the image classification results, which enhances the accuracy of moisture prediction based on classification confidence.

(2) $$Y=\sum_{i=0}^{n}Y_{i}*N_{i}$$

Here, *Y* represents the moisture content of the predicted sample, *Y_i_* represents the true *i*-sample moisture category label, *N_i_* represents the predicted *i*-sample category confidence, and *n* is the total number of sample categories entered for training; in this study, *n* = 9.

### 2.4. Evaluation

To better evaluate the predictive performance of the model on the moisture content of fresh tea leaves, this study used correlation coefficient (Rp), relative standard deviation (RPD), and root-mean-square error (RMSEP) as evaluation indicators for the model. The larger the Rp and RPD value, the smaller the RMSEP value, the better the predictive performance of the model [23]. Among these, RPD is the ratio of standard deviation to root mean square error of prediction, which can better reflect the performance of the model. When its value is greater than 2, it indicates that the model can well judge the degree of withering; when it is between 1 and 2, it indicates that the model has an average predictive ability for this parameter; when the RPD is less than 1, it indicates that the model cannot be used for this task. The calculation formulas for Rp, RMSEP, and RPD are shown in Equations (3)–(6).

(3) $$Rp=\sqrt{1-\frac{\sum_{i=1}^{n}{\left({\hat{y}}_{i}-y_{i}\right)}^{2}}{\sum_{i=1}^{n}{\left({\hat{y}}_{i}-\bar{y}\right)}^{2}}}$$

(4) $$RMSEP=\sqrt{\frac{\sum_{i=1}^{n}{\left(y_{i}-{\hat{y}}_{i}\right)}^{2}}{n}}$$

(5) $$RPD=\frac{SD}{RMSEP}$$

(6) $$SD=\sqrt{\frac{1}{N-1}\sum_{i=1}^{N}{\left(y_{i}-\bar{y}\right)}^{2}}$$

Among them, ${\hat{y}}_{i}$ represents the predicted value at time *i*, $y_{i}$ represents the true value at time *i*, and $\bar{y}$ represents the average value of 10 types of label data, with *SD* as the standard deviation.

### 2.5. Implementation Details

During the model training process, this study adopted the Pytorch learning framework and conducted an experiment in Windows 11 using the improved YOLOv8s as the benchmark model. The CPU was AMD Ryzen 5 7500F 6-Core Processor (3.70 GHz, 6 cores/12 threads, 32 MB), and the running memory was 32 GB; the GPU was NVIDIA GeForce RTX 4060 Ti 16G. During the training process, the hyperparameters for model training include a learning rate of 0.01, batch size of 16, epochs of 200, input image resolution of 224 × 224, momentum of 0.937, and a weight of 0.0005, using Adam optimizer.

## 3. Results and Discussion

### 3.1. Comparison Between Different Versions of YOLOv8

In this study, we conducted experiments on all five versions of the model, and the results are shown in Table 2. Among these, YOLOv8s exhibited a superior balance of performance characteristics. Specifically, YOLOv8s achieved an accuracy of 0.771, a value surpassed only by YOLOv8m, YOLOv8l, and YOLOv8x. It is crucial that YOLOv8 achieves this performance with a model size of only 4.856 MB, which is significantly smaller than the aforementioned versions. This reduced parameter count facilitates deployment in resource-constrained environments, such as mobile and embedded systems, where computational resources are limited.

Furthermore, YOLOv8s demonstrated impressive computational efficiency. While its inference time of 1.4 ms was slightly longer than the lightweight YOLOv8n (1.3 ms), YOLOv8s offered substantially improved accuracy. In addition, YOLOv8s achieved the fastest preprocessing time of 0.1 ms, further enhancing its real-time applicability. Based on these findings, YOLOv8s was selected as the foundational model for moisture content prediction, and its architecture was subsequently modified to improve predictive accuracy.

### 3.2. Comparative Experiments Before and After Model Optimization

**Figure 5:**
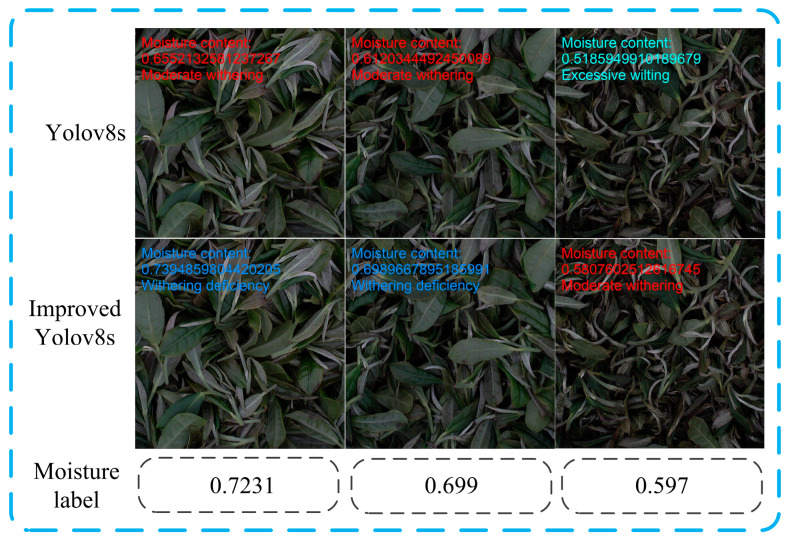
Comparison of model detection results before and after improvement.

To evaluate the superiority of the improved model, this study compared YOLOv8s with the improved model, and the results are shown in Table 3. The improved model reached a Top-1 accuracy of 0.927. This is a 0.156 accuracy increase over YOLOv8s. However, model parameters (Params) increased by 0.224 M, and floating-point operations (GFLOPs) increased by 1.2 G. Image processing speed rose by 0.2ms, and inference speed rose by 3.6ms. These results indicate that the model significantly improves accuracy while slightly increasing computation and detection time. This has great benefits for predicting moisture content based on confidence. Therefore, it is the best choice to increase calculation and detection time a little, greatly improving accuracy. Figure 5 illustrates the differences between the performance of the improved and initial models in identifying the degree of tea leaf wilting. This figure further highlights the superior performance of the improved model in real-time monitoring of tea leaf wilting.

### 3.3. Improve the Model to Predict Moisture Content and Wilting Degree

**Figure 6:**
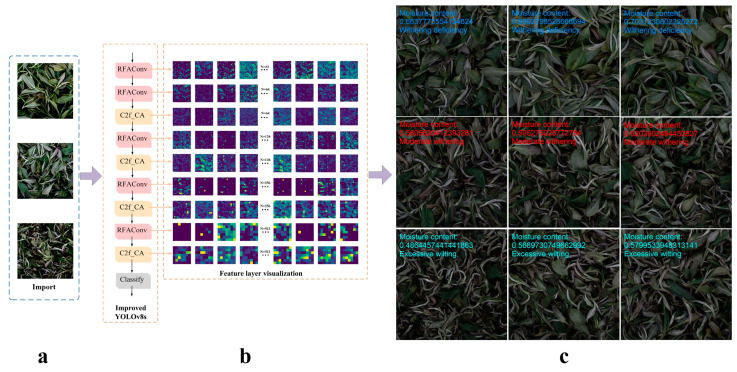
The entire process of improved model prediction. Model input (**a**). The network structure of the improved model and the visualization of feature maps at each layer (**b**). Identification results of the model (**c**).

Table 4 shows an example of the external test set. It shows the image category labels and moisture values in the training set, the prediction confidence of the three category samples in the external test set, and the predicted moisture content of fresh tea leaves in the external test set according to Equation (2). From Table 4, it can be seen that the errors between the predicted and true moisture values of the three types of external test sets are all less than 0.01, indicating that the model also achieves good detection results for untrained categories. In addition, the sum of the confidence levels of the three categories in the external test set in Table 4 is not completely equal to 1. This is because in this study, when predicting the external test set, the confidence level of a single image and the moisture value were only retained until eight decimal places, resulting in rounding errors. Figure 6 shows the entire process of the improved model prediction.

Table 5 shows the evaluation metrics for the relationship between the predicted results and the true values for the external validation set of the model. These data reveal that the RPD predicted by the model in this study is greater than 2, which well reflects the predictive performance of our model. However, compared with the other two categories, there is an obvious gap between the evaluation index base of category 9 in the external test set of the model. There may be two reasons for this: one is that the color and texture of fresh tea leaves change very little in continuous time, resulting in a larger prediction error of the model; the other is that a large error exists between the moisture labeled values in continuous time before and after category 9, leading to a bias toward the side with a high level of confidence in the computation process, which results in a larger prediction error.

### 3.4. Comparison of Moisture Content Prediction Using Different Models

**Figure 7:**
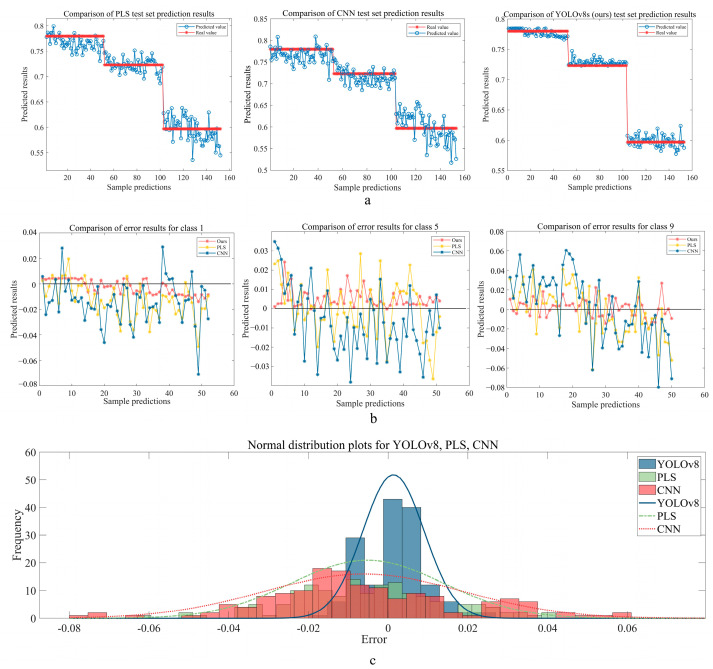
Prediction results of PLS, CNN, and YOLOv8 external test sets. (**a**) The distribution of predicted values and true values. (**b)** The distribution of errors. (**c**) Normal distribution.

Figure 7 shows the prediction results of PLS, CNN, and YOLOv8s on the external test set. Figure 7a presents the distribution of predicted values versus true values. It is evident that the predictions from our model are closest to the true values, demonstrating that our model has better generalization and robustness compared to PLS and CNN models. Figure 7b shows the prediction errors of the three models. From the error distribution, we can see that the error fluctuations between predicted and true values for the PLS and CNN models are much larger than those of our model. Although our model has notable outliers in Class 5 and Class 9, overall it achieves the best accuracy and is more suitable for online collection of wilted moisture content data from fresh tea leaf images. Figure 7c displays the normal distribution of prediction errors for the three models, helping us further explore the error intervals. Here, the horizontal axis represents the error range and the vertical axis represents the frequency of results within that range. From the normal distribution, it is clear that, compared to PLS and CNN, our model’s errors are more concentrated, mainly within the range of [−0.02, 0.02]. Additionally, the comparison of the three models’ Rp, RMSEP, and RPD values in Table 5 further confirms that our model performs well in predicting untrained categories.

## 4. Conclusions

This study addresses the need for detecting moisture content and determining wilting levels in fresh tea leaves. To improve the model’s prediction accuracy and reduce moisture content prediction errors, we introduced the RFAConv and C2f_CA modules. These additions significantly enhanced the model’s ability to extract and process features, resulting in a 0.156 improvement in detection accuracy. While this method increased the model’s parameters (Params) by 0.224 M and floating-point operations (GFLOPs) by 1.2 G, the average prediction time remained efficient at 5.3 ms. This balance ensures high detection accuracy while maintaining fast prediction speed.

In terms of experimental evaluation, this study achieved the best performance on the external test set 1, with Rp, RMSEP, and RPD values of 0.9983, 0.006278, and 39.2513, respectively. Further analysis revealed that the prediction errors were primarily within the range of [−0.02, 0.02], demonstrating high precision and stability. The results indicate that this model offers significant advantages in real-time detection of moisture content in fresh tea leaves and in determining their wilting levels. It effectively improves the accuracy of wilting degree judgment, providing reliable technical support for tea leaf quality assessment.

Although the proposed tea leaf withering degree detection model, though satisfactory, has limitations in accuracy, single-variety focus, and single-modal analysis. In future research, we will expand the diversity of research subjects and construct a multimodal perception framework. By deeply integrating and analyzing multidimensional data, the prediction accuracy and stability of the model can be further optimized.

## Figures and Tables

**Table 1:** YOLOv8s and improved YOLOv8s network layers.

Network Structure		Network Layer Before Optimization			Improved Network Layer		
Backbone	Layer number	Network Layer	Repeats	Kernels size	Network Layer	Repeats	Kernels size
	(1)	Conv	1	64	RFAConv	1	64
	(2)	Conv	1	128	RFAConv	1	128
	(3)	C2f	3	128	C2f_CA	3	128
	(4)	Conv	1	256	RFAConv	1	256
	(5)	C2f	6	256	C2f_CA	6	256
	(6)	Conv	1	512	RFAConv	1	512
	(7)	C2f	6	512	C2f_CA	6	512
	(8)	Conv	1	1024	RFAConv	1	1024
	(9)	C2f	3	1024	C2f_CA	3	1024
Head	Layer number	Network Layer	Repeats	Output	Network Layer	Repeats	Output
	(1)	Classify	1	10	Classify	1	10

**Table 2:** Detection results of the model.

Model	Acc	Para (MB)	GFLOPs (G)	Speed (ms)	
				Preprocessing	Inference
YOLOv8n	0.729	1.384	3.3	0.2	1.3
YOLOv8s	0.771	4.856	12.5	0.1	1.4
YOLOv8m	0.771	15.048	41.6	0.2	1.7
YOLOv8l	0.786	34.524	98.7	0.2	1.9
YOLOv8x	0.786	53.539	153.8	0.3	2.5

**Table 3:** Comparative experiments before and after model optimization.

Model	top1_acc	top5_acc:	Params (M)	GFLOPs (G)	Speed (ms)	
					Preprocessing	Inference
YOLOv8s	0.771	1	4.856	12.5	0.1	1.4
Ours	0.927	1	5.08	13.7	0.3	5

**Table 4:** Test set confidence and predicted value labels.

Training Set Category	Moisture Label	Test Set Confidence		
		1	5	9
0	0.7843	0.91888142	0	0
1	0.7714	0.08109235	0.00000007	0
2	0.763	0.00002619	0.00961427	0
3	0.7501	0.00000001	0.41952419	0
4	0.699	0	0.57085168	0.00000004
5	0.6654	0	0.00000980	0.00098720
6	0.6372	0	0	0.53591472
7	0.5671	0	0	0.45765832
8	0.5177	0	0	0.00543968
9	0.4863	0	0	0.00000003
Sum of Confidence		0.99999997	1.00000001	0.99999999
Predictive value		0.78325333	0.72105269	0.60449594
True value		0.7799	0.7231	0.597

**Table 5:** Evaluation indicators of different models for the test set prediction results.

	Ours			PLS			CNN		
	1	5	9	1	5	9	1	5	9
Rp	0.9983	0.993	0.9474	0.982	0.966	0.9488	0.9726	0.9277	0.905
RMSEP	0.006278	0.00694	0.018411	0.018615	0.014287	0.024974	0.022124	0.018567	0.03413
RPD	39.2513	20.1693	7.363	12.0667	7.974	6.4651	9.8045	5.5216	4.7987

## Data Availability

The original contributions presented in the study are included in the article, further inquiries can be directed to the corresponding author.
